# Three-Dimensional Aggregates Enhance the Therapeutic Effects of Adipose Mesenchymal Stem Cells for Ischemia-Reperfusion Induced Kidney Injury in Rats

**DOI:** 10.1155/2016/9062638

**Published:** 2015-11-16

**Authors:** Xiaozhi Zhao, Xuefeng Qiu, Yanting Zhang, Shiwei Zhang, Xiaoping Gu, Hongqian Guo

**Affiliations:** ^1^Department of Urology, Affiliated Drum Tower Hospital, School of Medicine, Nanjing University, Nanjing 210008, China; ^2^Institute of Urology, Nanjing University, Nanjing 210093, China; ^3^State Key Laboratory of Pharmaceutical Biotechnology, Nanjing University, Nanjing 210093, China; ^4^Department of Anesthesiology, Affiliated Drum Tower Hospital, School of Medicine, Nanjing University, Nanjing 210008, China

## Abstract

It has been shown that administration of adipose derived mesenchymal stem cells (AdMSCs) enhanced structural and functional recovery of renal ischemia-reperfusion (IR) injury. Low engraftment of stem cells, however, limits the therapeutic effects of AdMSCs. The present study was designed to enhance the therapeutic effects of AdMSCs by delivering AdMSCs in a three-dimensional (3D) aggregates form. Microwell was used to produce 3D AdMSCs aggregates. In vitro data indicated that AdMSCs in 3D aggregates were less susceptible to oxidative and hypoxia stress induced by 200 *μ*M peroxide and hypoxia/reoxygenation, respectively, compared with those cultured in two-dimensional (2D) monolayer. Furthermore, AdMSCs in 3D aggregates secreted more proangiogenic factors than those cultured in 2D monolayer. 2D AdMSCs or 3D AdMSCs aggregates were injected into renal cortex immediately after induction of renal IR injury. In vivo data revealed that 3D aggregates enhanced the effects of AdMSCs in recovering function and structure after renal IR injury. Improved grafted AdMSCs were observed in kidney injected with 3D aggregates compared with AdMSCs cultured in 2D monolayer. Our results demonstrated that 3D AdMSCs aggregated produced by microwell enhanced the retention and therapeutic effects of AdMSCs for renal IR injury.

## 1. Introduction

Mesenchymal stem cells (MSCs) are multipotent progenitor cells which could be isolated from several tissues, in particular bone marrow and adipose tissue [1, 2]. The ease of isolation and expansion in culture, the immunomodulatory properties, and the multilineage differentiation potential make MSCs promising resource for cell therapy. Several studies have demonstrated that administration of MSCs enhanced structural and functional recovery of renal ischemia-reperfusion (IR) injury [3–5]. The beneficial effect of injected MSCs was suggested to be mainly related to the secretion of cytokines from transplanted MSCs, which is defined as paracrine effect [4, 6].

Although the therapeutic effects of MSCs for renal IR injury have been well described, a lack of initial engraftment and retention due to early cell death is a major roadblock to achieving clinical significance [7]. This might be due to the “harsh environment” including oxidative stress, hypoxia in the injured kidney. Several strategies have been developed to improve the survival of engrafted stem cells in ischemic kidney. These strategies include genetic modification of stem cells [8, 9], pretreatment of MSCs [7, 10], and the use of tissue engineering scaffolds [11]. Despite the improved survival of stem cells in ischemic kidney, the strategies still have problems for further clinical application.

Recently, many researchers demonstrated that delivering MSCs in an aggregate form could serve as an effective method to solve these problems. It has been shown that three-dimensional (3D) aggregates enhanced the survival of engrafted cells as well as the microenvironment of injured organs. For example, delivery of cardiac progenitor cells in the form of 3D aggregates improved in vivo survival of implanted cells in cardiac ischemia-reperfusion injury [12]. Following intramuscular transplantation to ischemic limbs, 3D MSCs aggregates showed improved cell survival and limb survival [13]. However, few researches have focused on the application of 3D MSCs aggregates in stem cell-base therapy of renal IR injury. Several 3D cell culture techniques including porous scaffolds, hydrogel, and cellular aggregates have been developed. Among them, cellular aggregates have drawn rising attention because they are free of exogenous biomaterials [14]. Microwell has been reported as an effective technique to produce scale cell aggregates [12, 15]. The present study was designed to examine the therapeutic effects of 3D adipose derived MSCs (AdMSCs) aggregates produced by microwell for renal IR injury using a rat model. We hypothesize that delivery of AdMSCs in the form of aggregates could enhance the survival of AdMSCs in injured kidney and therefore improve the therapeutic effects of AdMSCs. To the best of our knowledge, this is the first study designed to explore the feasibility of using 3D AdMSCs aggregates to enhance the therapeutic effects of AdMSCs for renal IR injury.

## 2. Materials and Methods

### 2.1. Cell Culture

AdMSCs were isolated from paratesticular fat of Sprague-Dawley rats and cultured according to our previously described protocol [16, 17]. Briefly, adipose tissue was minced into small pieces and then incubated with 0.075% type I collagenase (Sigma-Aldrich, St. Louis, MO, USA) for 1 h at 37°C. After centrifuging at 220 g for 10 min, the top lipid layer was removed and the remaining cells were suspended in Dulbecco's modified Eagle's medium (DMEM, Life Technologies, Shanghai, China) supplemented with 10% fetal bovine serum (FBS, Life Technologies), and plated at a density of 1 × 10^6^ cells in a 10 cm dish. AdMSCs were passed under the same conditions through no more than five passages before being used for assays. All procedures were approved by Institutional Animal Care and Use Committee of Nanjing University.

### 2.2. Microwell Assembly and Generation of AdMSCs Aggregates

Microwells were generated using micromolding on UV-photocrosslinkable polyethylene glycol dimethacrylate (PEG, MW = 1000, 20% in PBS) (Sigma-Aldrich) with 1% photoinitiator 2-hydroxy-1-[4-(hydroxyethoxy) phenyl]-2-methyl-L-propanone (Irgacure D2959, Ciba Specialty Chemicals Inc., Florham Park, NJ, USA) according to the previously described protocol [12, 15]. Briefly, PEG macromer solution was pipette on glass slide coated with 3-(trimethoxysilyl) propylmethacrylate (TMSPMA) (Sigma-Aldrich). Thereafter, a patterned PDMS stamp was placed on PEG solution to make PEG solution distribute evenly between PDMS stamp and TMSPMA coated glass slide. After 10 seconds of irradiation with UV (*l* = 350–500 nm, 10 mW/cm^2^; OmniCure Series 2000 curing station, EXFO, Mississauga, Canada), the PEG was photocrosslinked with microwells on it. For generation of AdMSCs aggregation, AdMSCs were seeded onto microwells by using a previously developed method [18]. Briefly, AdMSCs suspension (1 × 10^6^ cells/mL) was pipetted on the surface of the microwell array evenly (100 *μ*L/cm^2^). Two hours later, the array was immersed in media in a culture dish. AdMSCs formed 3D aggregates in microwell. A schematic summarizing the protocol is shown in Figure S.1 (see Supplementary Material available online at http://dx.doi.org/10.1155/2016/9062638).

### 2.3. Hydrogen Peroxide (H_2_O_2_) and Hypoxia/Reoxygenation Treatment

To determine the sensitivity of the cells to stress including oxidative stress and hypoxia, AdMSCs cultured in 2D monolayer or in 3D aggregates were treated with 200 *μ*M H_2_O_2_ or hypoxia followed reoxygenation. Briefly, cells were incubated with culture medium containing 200 *μ*M H_2_O_2_ or vehicle for 2 hours to mimic oxidative stress. For the induction of hypoxia/reoxygenation, cells were subjected to hypoxia for 24 h followed by 2 hours of reoxygenation. Hypoxia (2% O_2_) was achieved by using a multigas incubator (Sanyo, Pfaffenhofen, German) that maintained a gas mixture of 2% O_2_, 5% CO_2_, and 93% N_2_ at 37°C. Cells cultured under regular culture conditions for the same period of time were set as control. Viability of cells was determined as described below.

### 2.4. Cell Viability

A rapid, simultaneous double-staining procedure using fluorescein diacetate (FDA) and propidium iodide (PI) (Sigma-Aldrich) was used in the determination of cell viability [19]. In brief, cells were stained with 5 *μ*g/mL PI and 4 *μ*g/mL FDA and observed under the fluorescent microscopy with an appropriate barrier filter set.

### 2.5. Real Time Polymerase Chain Reaction (PCR)

Gene expression of extracellular matrix (ECM) and proangiogenic growth factors were determined by real time PCR. Briefly, total RNA was isolated from cells using TRIzol Reagent (Life Technologies, Shanghai, China). RNA was reverse-transcribed (RT) to cDNA by using a commercial available transcription Kit (Applied Biosystems, Carlsbad, CA, USA). Quantitative RT-PCR was performed using the Power SYBR Green PCR Master Mix (Life Technologies). All experiments were performed in triplicate for each sample and each gene. The primer sequence was listed in Table S.1.

### 2.6. Immunofluorescent Analysis

After rinsed with PBS and fixed with 4% paraformaldehyde for 10 min, cells were rinsed with PBS and permeabilized with 0.05% triton X-100 for 10 min. After incubated with 5% horse serum for 30 min and then with primary antibody over night at 4°C, the cells were incubated with secondary antibodies for 1 h at room temperature. The primary antibodies included anti-fibronectin (1 : 100, Santa Cruz Biotechnology, Santa Cruz, CA, USA), anti-laminin (1 : 100, Santa Cruz Biotechnology). The secondary antibody is Alexa-594-conjugated secondary antibodies (1 : 500; Life Technologies). Nuclei were stained with 4′,6-diamidino-2-phenylindole (DAPI; Life Technologies).

### 2.7. Animals and Treatment

All experimental protocols conducted on animals were performed in accordance with the standards established by the Institution Animal Care and Use Committee at Nanjing University. Male Sprague-Dawley rats weighing 200–250 g were housed in stainless steel cages and given free access to food and water. Rats were randomly divided into four groups. The sham group received sham surgery (*n* = 6); the IR group received injection of PBS immediately after the induction of renal IR (*n* = 10); the IR + AdMSCs group received injection of 2D AdMSCs after the induction of renal IR (*n* = 10); the IR + aggregates group received injection of 3D AdMSCs aggregates after the induction of renal IR (*n* = 10). Rats were euthanized 24 h after surgery. Blood samples were collected for measurement of renal function while kidneys were collected for molecular analysis.

### 2.8. Rat Model of Renal IR Injury

Rats were anesthetized intraperitoneally with a mixture of ketamine (100 mg/kg) and midazolam (5 mg/kg). After anesthetization, Rats were subjected to renal IR injury as previously described [20]. Briefly, after abdominal laparotomy, right kidney was exposed and removed. Left renal pedicle was exposed and clamped with vascular clamp for 50 min to induce ischemia. After clamp, vascular clamp was removed to induce reperfusion and the incision was closed in 2 layers. Sham-operated control animals underwent right nephrectomy only.

### 2.9. Cell Labeling and Injection

For the cell tracking, AdMSCs were labeled with EdU (Life Technologies, Shanghai, China) for 48 hr according to the instructions provided by the manufacturer. Since it takes about 24 h for AdMSCs to form 3D aggregates in microwell, a total of 1 × 10^6^ EdU labeled AdMSCs were seeded onto 2D cultural dish or microwell. Twenty-four hours later, cells or aggregates were isolated from 2D cultural dish or microwell for injection. AdMSCs or 3D AdMSCs aggregates in 90 *μ*L PBS were injected into the kidney cortex using a 28 G needle (three injections: poles and middle area) according to a previously published protocol [11].

### 2.10. Measurement of Renal Function

Serum was separated by centrifuging blood samples and stored at −80°C until analysis of blood serum urea nitrogen (BUN) and urine creatinine (Cr). The concentrations of BUN and Cr were assessed in duplicated with a commercially available assay kit (BioAssay System, Hayward, CA, USA) according to the instructions.

### 2.11. Histological Analysis

Middle part of kidney was fixed in 4% formaldehyde, dehydrated, and paraffin embedded. Tissue sections (5 *μ*m) were stained with hematoxylin and eosin (HE). Kidney sections were examined in a blinded manner and scored to evaluate the degree of injury. The score reflected the grading of tubular necrosis, cast formation, tubular dilation, and loss of brush border in 10 randomly selected, nonoverlapping fields (200x) as follows: 0: none; 1: ≤10%; 2: 11 to 25%; 3: 26 to 45%; 4: 46 to 75%; and 5: ≥76% [20, 21].

### 2.12. Detection of Apoptosis

Quantitative determination of apoptosis in kidney sections was assessed by a terminal transferase-mediated dUTP nick-end labeling (TUNEL) assay using an in situ cell death detection kit (Roche, Basel, Switzerland). After dewaxing and rehydration, the tissue sections were permeabilized with 0.1% Triton X-100 for 10 min. Incubation with label solution was used to detect the apoptotic cells according to the instructions. Apoptotic score was achieved by counting the number of positive nuclei in 10 random fields.

### 2.13. Detection of EdU Positive Cells

Quantitative determination of EdU positive cells in kidney sections was assessed. Briefly, after dewaxing and rehydration, the tissue sections were permeabilized with 0.1% Triton X-100 for 10 min. Incubation with Click-iT^@^ reaction cocktails was used to detect the apoptotic cells according to the instructions (Invitrogen). Cell engraftment was determined by counting the number of positive nuclei in 10 random fields.

### 2.14. Statistical Analysis

All statistical analysis was performed using Prism 4 (GraphPad Software, San Diego, CA, USA). One-way analysis of variance (ANOVA) followed by the Tukey-Kramer test for post hoc comparisons was used for analyzing difference between groups. Statistical significance was set at *P* < 0.05.

## 3. Results

### 3.1. Construction of 3D AdMSCs Aggregates

**Figure 1:**
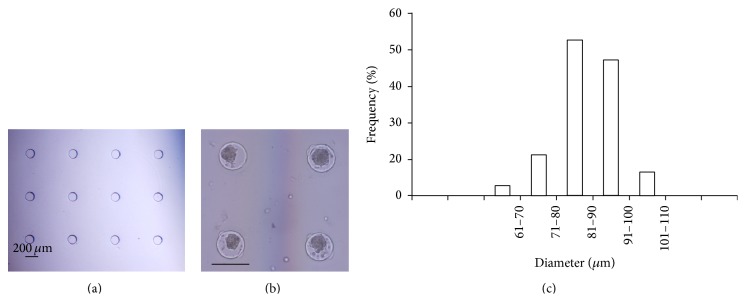
Microwells for culturing AdMSCs derived aggregates. (a) Phase contrast image of microwell. (b) Phase contrast image of AdMSCs aggregates formed in microwell. Black bar indicates 200 *μ*m. (c) Frequency distribution of diameter of aggregates formed in microwell.

AdMSCs aggregated and formed well defined 3D aggregates 24 h following seeding. The AdMSC aggregates did not disassemble for culture time up to 14 days (data not shown). The size of AdMSCs aggregates was homogenous with low variation (Figure 1(c)).

### 3.2. Characterization of 3D AdMSCs Aggregates

**Figure 2:**
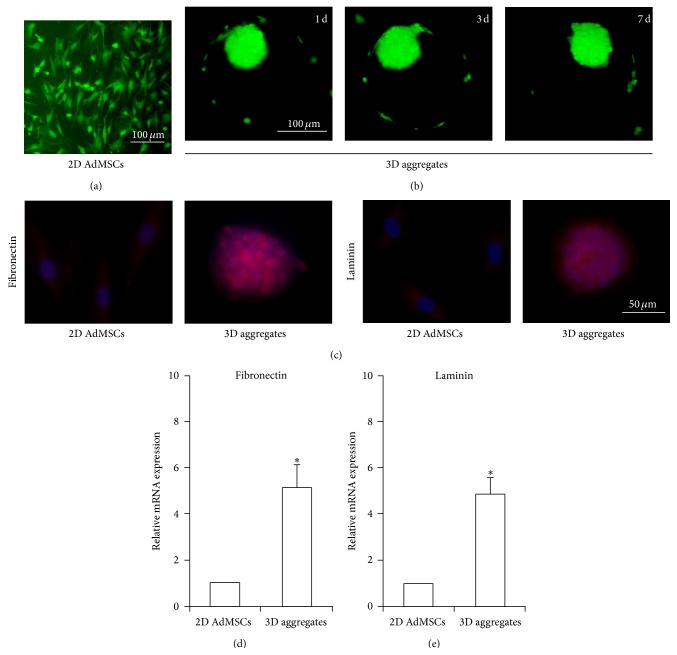
Characters of 3D AdMSCs aggregates. (a) Viability of AdMSCs cultured in 2D monolayer. (b) Viability of 3D AdMSCs aggregates 1 d, 3 d, and 7 d after seeding in microwell. Live or dead cells were stained with FDA (live, green) PI (dead, red). (c) Fluorescent images of 2D AdMSCs or 3D aggregates stained with fibronectin and laminin. (d) mRNA expression of fibronectin and laminin in 2D AdMSCs or 3D aggregates. ^*∗*^ *P* < 0.05 compared with 2D AdMSCs.

As shown in Figure 2(b), most cells in 3D aggregates were positive for FDA and negative for PI staining, suggesting high viability in the aggregates for at least 7 days (Figures 2(a) and 2(b)). 3D AdMSCs aggregates showed well-preserved ECM compared to the 2D AdMSCs. After culture in microwell for 7 days, cells expressed significantly increased the level of fibronectin and laminin (Figures 2(c)–2(e)) compared with 2D cells, partly indicating the significantly increased secretion of ECM in 3D AdMSCs aggregates.

### 3.3. 3D AdMSCs Aggregates Are Less Susceptible to Oxidative and Hypoxia Stress

**Figure 3:**
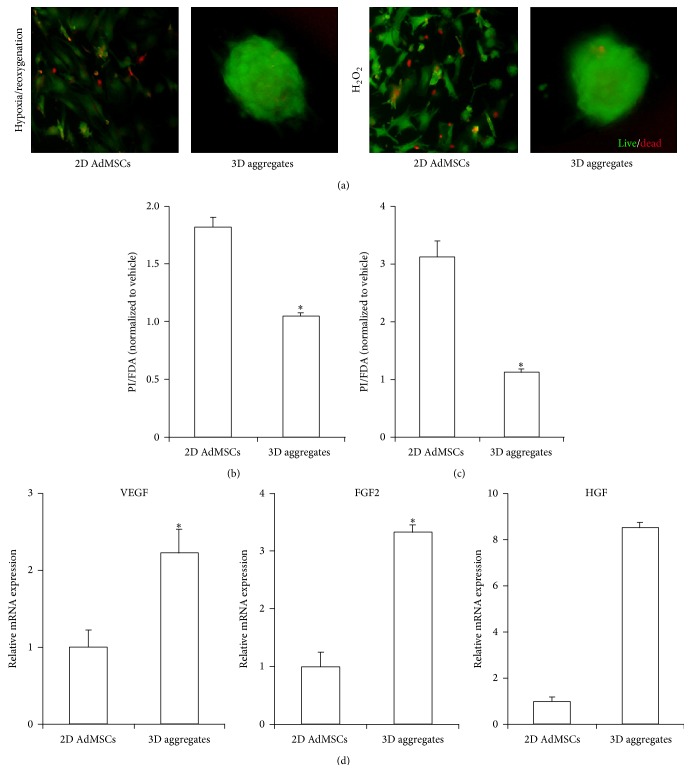
3D aggregates decrease the susceptibility of AdMSCs to stress and enhance the expression of proangiogenic factors in AdMSCs. (a) Representative FDA/PI stained images of 2D AdMSCs or 3D aggregates subjected to hypoxia/reoxygenation or 200 *μ*M-H_2_O_2_ treatment. (b) Quantification of dead cells in 2D AdMSCs or 3D aggregates subjected to hypoxia/reoxygenation treatment using PI/FDA ratio. Data were normalized to the vehicle group of 2D monolayer culture. (c) Quantification of dead cells in 2D AdMSCs or 3D aggregates subjected to 200 *μ*M-H_2_O_2_ treatment using PI/FDA ratio. Data were normalized to the vehicle group of 2D monolayer culture. (d) Relative mRNA expression of VEGF, FGF2, and HGF in 2D AdMSCs and 3D aggregates. ^*∗*^ *P* < 0.05 compared with 2D AdMSCs.

To verify that cells in 3D aggregates enhance protection against hypoxia, AdMSCs in either 2D monolayer culture or 3D aggregates were subjected to 24 h of hypoxia followed by 2 h reoxygenation. As shown in Figures 3(a) and 3(b), cells grown in 3D aggregates were less susceptible to hypoxia/reoxygenation-induced stress compared to those cultured in 2D monolayer as determined by PI/FDA ratio. In addition, we examined the response of 2D AdMSCs or 3D aggregates to oxidative stress mimicked by 200 *μ*M H_2_O_2_. Similar to results obtained following hypoxia/reoxygenation, reduced cell death, as determined by PI/FDA, was observed in 3D aggregates (Figures 3(a) and 3(c)).

### 3.4. Enhanced Secretion of Proangiogenic Growth Factors from 3D AdMSCs Aggregates

Real time PCR was used to detect the mRNA expression of proangiogenic factors in 2D AdMSCs or 3D aggregates. 3D aggregates showed considerable expressions of proangiogenic growth factors. As shown in Figure 3(d), the expressions of vascular endothelial growth factor (VEGF), fibroblast growth factor-2 (FGF2), and hepatocyte growth factor (HGF) were much greater than those of 2D AdMSCs monolayer (Figure 3(d)).

### 3.5. 3D AdMSCs Aggregates Preserve Renal Function and Renal Histology

**Figure 4:**
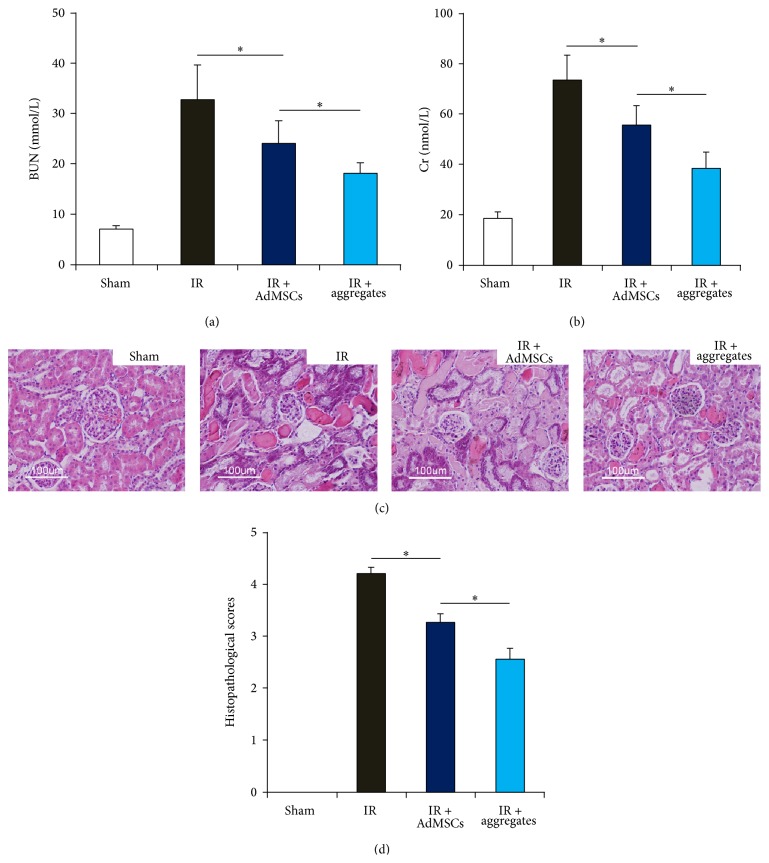
2D AdMSCs or 3D aggregate preserves renal function and histology after IR. ((a)-(b)) Circulating levels of urea nitrogen (BUN) and creatinine (Cr) in different experimental groups (*n* = 6 in each group). (c) Representative images of HE staining of kidney sections in each experimental group, showing significantly higher degree of tubular damage including tubular necrosis, cast formation, dilatation of tubules, and loss of brush border in IR group. (d) Results of total histopathological scores reflecting tubular damage in each group. ^*∗*^ *P* < 0.05.

Serum levels of BUN and Cr were selected to determine the renal function. As shown in Figures 4(a) and 4(b), both BUN and Cr levels were significantly higher in IR group than sham group. Injection of AdMSCs, either 2D monolayer culture or 3D aggregates, showed positive effects in preserving renal function, reflected by significantly reduced BUN and Cr levels compared to the IR group. More importantly, the levels of BUN and Cr in IR + aggregates group were significantly lower compared with those in 2D AdMSCs group. These findings implicated that 3D aggregates showed significantly improved protective effects compared with 2D AdMSCs.

To determine the effect of AdMSCs on IR-induced renal injury, a histological scoring system bases on the typical microscopic features of acute tubular damage was adopted (Figure 4(c)). At 24 hours after the IR procedure, the injury score was highest in IR group, significantly higher than sham group. The injury scores in IR + AdMSCs and IR + aggregates group were significantly reduced compared with IR group. The injury score in IR + aggregates group was significantly lower compared with that in IR + AdMSCs group (Figure 4(d)). These findings suggested that 3D aggregates offered more protection than 2D AdMSCs.

### 3.6. 3D Aggregates Reduced Apoptosis in Kidney after IR Procedure

**Figure 5:**
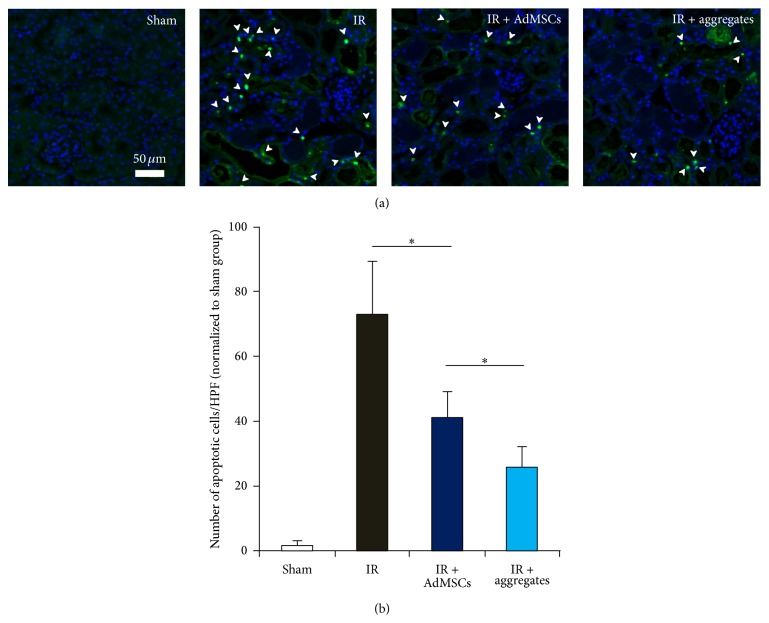
2D AdMSCs or 3D aggregate reduces tubular apoptosis after IR. (a) Representative images of TUNEL staining of kidney sections in each experimental group. White arrow indicated apoptotic cells in tubules. (b) Results of the number of apoptotic cells in kidney sections in each group. ^*∗*^ *P* < 0.05.

To investigate IR associated apoptotic cells, we measured TUNEL positive cells in kidney tissues. As shown in Figure 5, 24 hours after IR, no apoptotic cells were observed in the kidney from sham group. The number of apoptotic cells increased significantly in IR group compared with sham group. In contrast, tissues from AdMSCs and aggregates groups contain a significantly smaller number of TUNEL positive apoptotic cells. Furthermore, the number of apoptotic cells in 3D aggregates group reduced significantly in 3D aggregates group compared with that in 2D AdMSCs.

### 3.7. 3D Aggregates Improved In Vivo Survival of AdMSCs

**Figure 6:**
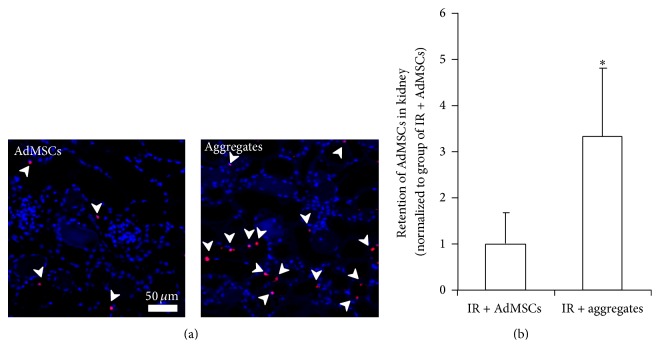
Retention of AdMSCs in kidney. (a) Representative images of EdU-labeled AdMSCs (indicated by white arrow) identified in kidney of rats underwent injection of 2D AdMSCs or 3D aggregates. (b) Results of EdU positive AdMSCs quantification in each high power field (HPF). Data were normalized to IR + AdMSCs group. ^*∗*^ *P* < 0.05 compared to IR + AdMSCs group.

The number of EdU positive AdMSCs in the renal section was counted to determine cell engraftment after injection. As shown in Figure 6, the number of EdU positive cells in the renal section from 3D aggregates group was significantly greater compared with that in 2D monolayer, suggesting that the form of 3D aggregates exhibit greater survival when implanted in vivo following renal IR injury.

## 4. Discussion

Our results show for the first time that microwell produced 3D AdMSCs aggregates improved renal function by suppressing IR-induced elevation of BUN and Cr, restoring IR-damaged renal histological structures, and decreasing tubular cell apoptosis compared with 2D AdMSCs monolayer.

Renal IR injury is one of serious and common diseases with high morbidity and mortality in clinical nephropathy. It is often an unavoidable side effect in renal transplantation and frequently occurs as a result of shock or surgery [22]. Unfortunately, innovative interventions beyond supportive therapy are not yet available. Therefore, it is urgent to develop a new and effective approach for renal IR injury repair.

In the last several years, many studies have shown that transplantation of MSCs is an effective strategy for renal IR injury [5, 23]. MSCs isolated from different tissues including bone marrow [24], adipose [25, 26], Wharton's jelly [27], fetal membrane [28], and umbilical cord [29] have been transplanted to protect against renal IR injury. Among them, AdMSCs were one of the most promising seeding cells because of their abundance in existence, ease in harvesting. Several comparison studies have shown that AdMSCs are similar in cell surface expression profiles, differentiation potential and therapeutic efficacy with MSCs derived from bone marrow [30–32]. Most importantly, sufficient number of AdMSCs for clinical application could be obtained with minimal side effects under local anesthesia [33], making AdMSC an alternative cell source for repair of renal IR injury. Despite the advance of AdMSCs-based therapy for renal IR injury, low retention and extensive early death of grafted cells had been one of the remaining problems.

Recently, delivering MSCs in a 3D form has been demonstrated to be an effective strategy to enhance the survival and therapeutic effects of stem cells [14]. Compared to traditional 2D substrates, 3D culture mimics the in vivo microenvironment and maximizes the cell-cell communication required for stem cell function. In the present study, 3D AdMSCs aggregates were used to promote the survival rate of grafted AdMSCs. Several approaches such as porous scaffolds, hydrogel, and cellular aggregates have been successfully applied to provide 3D culture environment for a variety of cells types, including MSCs [14]. Among these techniques, cellular aggregates have drawn increasing attention because they are free of exogenous biomaterials that may cause untoward responses upon cell transplantation [34, 35]. As a main technique of cellular aggregates, hanging drop has been widely used for stem cell culture [14]. However, hanging drop is ineffective in producing large scale of aggregates. In the present study, we used PEG derived microwell array as a 3D culture system to form AdMSCs aggregates. As shown in Figure 1, the size of AdMSCs aggregates was homogenous with low variation. Most importantly, microwell is effective in producing large scale of AdMSCs aggregates compared with hanging drop. Furthermore, medium change for microwell array is as easy as for regular 2D cell culture, which makes it feasible for longer in vitro culture. Additionally, many in vitro tests can be directly performed within microwell array without having to transfer cells to another container.

In the present study, less susceptibility to oxidative and hypoxia stress was observed in 3D AdMSCs aggregates compared with AdMSCs cultured in 2D monolayer. Being consistent with the in vitro data, significantly increased grafted AdMSCs were also observed in IR kidney. The protective effects of 3D aggregates against stress, such as oxidative stress and hypoxia, might be due to the preconditioning of AdMSCs in the 3D microenvironment [14]. It has been described that 3D aggregates structure could create a heterogeneous microenvironment which provides a size-dependent gradient of nutrients, oxygen, and cytokines. Oxygen is suggested to be a primary factor that affects the biological properties of MSCs within the aggregates. 3D AdMSCs aggregates have been demonstrated to provide a mild hypoxic environment and promote the secretion of hypoxia-inducible factor (HIF) [13, 36]. It has been demonstrated that stem cells underwent apoptosis immediately after exposing to harsh environment including oxidative stress, hypoxia. Transplantation of 3D aggregates may inhibit apoptosis by preconditioning AdMSCs to hypoxic microenvironment and by stimulating expression of hypoxia-induced survival factors such as HIF.

It is generally accepted that the main therapeutic mechanism of AdMSCs for IR-induced renal injury is through the expressions of cytokine, which is defined as paracrine effect. Increased grafted stem cells are responsible for the enhanced therapeutic effects of AdMSCs. Furthermore, it has been demonstrated that 3D aggregates upregulated secretion of proangiogenic factors such as VEGF, FGF-2, and HGF from MSCs, which is suggested to be induced by a mild hypoxic environment in 3D aggregates [13, 36]. From our in vitro results, the secretion of proangiogenic factors was promoted in 3D AdMSCs aggregates, which is consistent with previously reported results [13]. In addition, transplantation of 3D AdMSCs aggregates to ischemic kidney inhibited tubular apoptosis when compared with 2D AdMSCs grown in monolayer. This may due to the upregulated expressions of proangiogenic factors, which could protect tubular epithelium against IR-induced injury.

Our study has some limitations. First, the retention of AdMSCs aggregates was observed only 24 h followed IR procedure. It would be interesting to detect the retention of AdMSCs aggregates at longer time points. We have started experiments aimed at identifying retention of AdMSCs in kidney at longer time points after IR injury. Second, 3D AdMSCs aggregates were delivered directly into the injured kidney. Despite the therapeutic effects, this delivery method is not suitable in clinical application. Further study would be conducted to investigate the effect of AdMSCs aggregates injected intravenously.

## 5. Conclusion

Our data provides for the first time that 3D AdMSCs aggregates possess survival benefits when implanted into rat kidney following IR injury. Microwell is effective in producing scale of 3D AdMSCs aggregates, which could be utilized to advance the efficacy of AdMSCs therapy for renal IR injury.

## Supplementary Material

Schematic of the processes to fabricate microwell and 3D aggregates, as well as the primer sequences for real-time PCR analysis are shown in supplementary materials.
