# Supplementary material for: Three-Dimensional Aggregates Enhance the Therapeutic Effects of Adipose Mesenchymal Stem Cells for Ischemia-Reperfusion Induced Kidney Injury in Rats
Source: Stem Cells Int. 2015 Nov 16;2016:9062638. doi: 10.1155/2016/9062638 (PMC4663369; doi:10.1155/2016/9062638)
Supplement: Supplementary file 1 — Schematic of the processes to fabricate microwell and 3D aggregates, as well as the primer sequences for real-time PCR analysis are shown in supplementary materials. [file 9062638.f1.docx]

**Supplementary Materials**


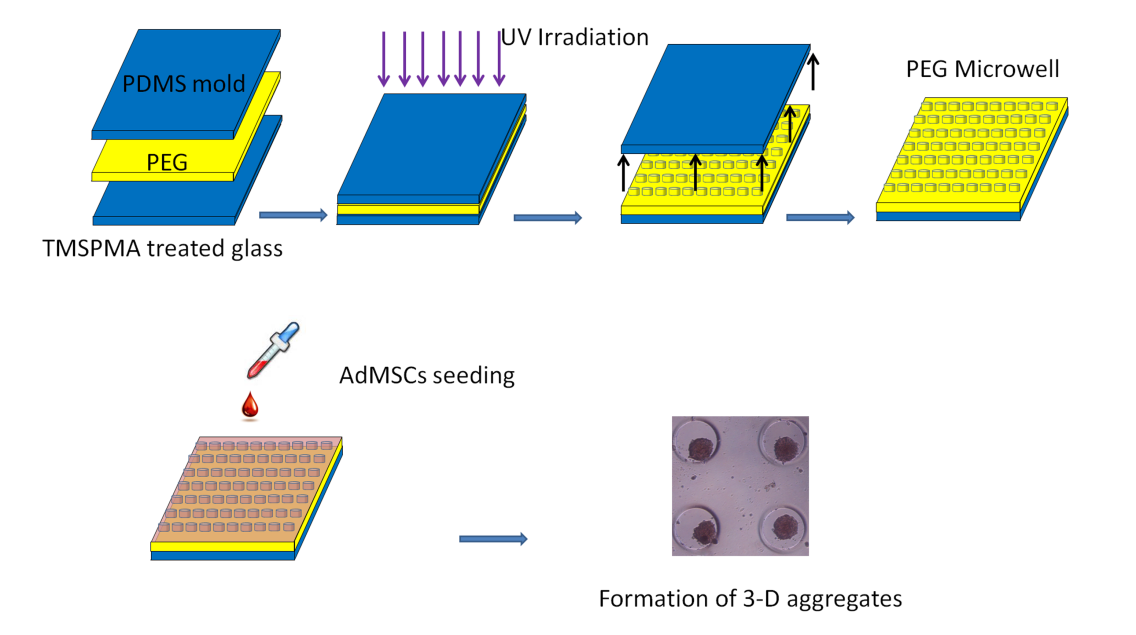


Figure.S.1 Schematic of the process to fabricate microwell and 3-D AdMSCs aggregates.

Table.S.1 Primer sequences for real-time PCR analysis

| Target gene |  | Primer sequences |
| --- | --- | --- |
| VEGF | Forward  Reverse | 5'- ATCTTCAAGCCGTCCTGTGT -3'  5'- GTGGTCACTTACTTTTCTGGCT -3' |
| FGF2 | Forward  Reverse | 5'- GAGAAGAGCGACCCACACG -3'  5'- ACACACTTAGAAGCCAGCA -3' |
| HGF | Forward  Reverse | 5'- AGAGAGGCGAGGAGAAACG -3'  5'- TCCACGACCAGGAACAATG -3' |
| Fibronectin | Forward  Reverse | 5'- GTGAAGAACGAGGAGGATGTG -3'  5'- GTGATGGCGGATGATGTAGC -3' |
| Laminin | Forward  Reverse | 5'- GTGAAGAACGAGGAGGATGTG -3'  5'- TCCTCCTGGCATCTGCTGACTC -3' |
| GAPDH | Forward  Reverse | 5'-CAACTCCCTCAAGATTGTCAGCAA-3'  5'-GGCATGGACTGTGGTCATGA-3' |
